# Polymorphous Adenocarcinoma: High-Grade Transformation With Immunohistochemical Workup

**DOI:** 10.7759/cureus.23639

**Published:** 2022-03-30

**Authors:** Sudhakara Muniswammappa, Radhika Bavle, Soumya Makarla, Reshma Venugopal

**Affiliations:** 1 Department of Oral and Maxillofacial Pathology, Krishnadevaraya College of Dental Sciences and Hospital, Bangalore, IND

**Keywords:** pac, high grade transformation (hgt), primary high grade transformation, invasive pac, comedo necrosis, minor salivary gland, buccal mucosa

## Abstract

Intraoral tumours associated with mucosa are commonly oral squamous cell carcinomas followed by minor salivary gland carcinomas, the commonest being mucoepidermoid carcinoma, adenoid cystic carcinoma, and polymorphous adenocarcinoma (PAC). PAC is the second most common malignant tumour that is found in the minor salivary glands of the oral cavity and rarely exhibits high-grade transformation (HGT). We report a case of a 50-year-old woman with a tumour on her buccal mucosa for six months. Histopathologically, the tumour showed more than 15 histopathological patterns with areas of HGT. The high-grade transformed areas predominantly showed solid patterns, increased mitosis, necrosis, vascular invasion, and perineural invasion. An immunohistochemical (IHC) panel inclusive of p63, SOX-10, S-100, calponin, vimentin, and Ki-67 was done to evaluate the tumour and grade PAC. The Ki-67 index was around 25%-30%, confirming the diagnosis of PAC-HGT. This might be the first case of primary PAC-HGT seen on the buccal mucosa on initial clinical presentation.

## Introduction

Polymorphous adenocarcinoma (PAC) is a salivary gland tumour that has seen an interesting journey in nomenclature and behaviour from its conception as a tumour way back in 1984 until the WHO 2017 classification and categorization of salivary gland tumours. PAC was classically a low-grade carcinoma called polymorphous low-grade adenocarcinoma (PLGA). Innumerable descriptions of this tumour have been woven together with robust data on histopathology and behaviour and its behaviour has become questionable in the past decades. Cases with high-grade transformation (HGT) or dedifferentiation in PAC have been reported. HGT in salivary gland tumours, though rare, does occur. It has been reported in other salivary gland carcinomas like acinic cell carcinoma (AciCC), adenoid cystic carcinoma (AdCC), epithelial-myoepithelial carcinoma (Epi-myoepi CA), secretory carcinoma (SC), and mucoepidermoid carcinoma (MEC) [1]. English literature reports only about seven cases of PAC with HGT, which is a rare finding. De novo HGT is the rarest, as most of it is accounted for in cases of recurrence, post-radiation, and post-treatment [2]. The present case highlights the occurrence of an HGT-PAC on initial presentation, seen on the buccal mucosa close to the retromolar area, which might be the first case report of HGT-PAC on the buccal mucosa.

## Case presentation

A 50-year-old Indian woman presented with a small swelling, approximately one cm in diameter on the buccal mucosa in relation to the maxillary left molars. When the patient first presented to the clinic, she stated that the lesion had been noticed around eight months prior and was an asymptomatic swelling. The patient presented again after a fortnight to the clinic with complaints of an increase in the size of the swelling and ulceration, which may be due to a cheek bite.

**Figure 1:**
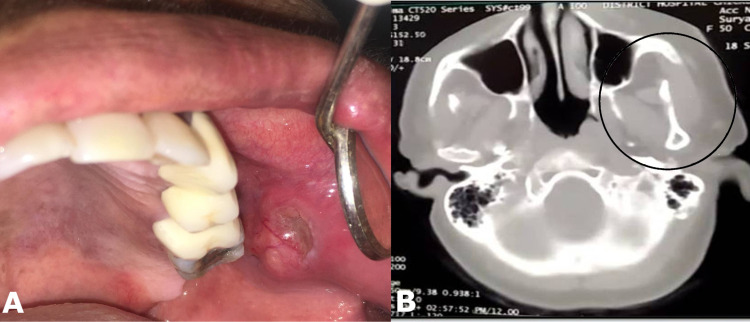
(A) Clinical image showing a nodular cystic swelling of around 2 cm opposite to left maxillary second premolar and first molar teeth region on the buccal mucosa. (B) Computed tomogram (CT) scan showing normal maxillary bone and maxillary antral areas with no bony involvement by the tumour (area highlighted by a circle).

On inspection, a spherical soft tissue mass of approximately one-and-half cm in diameter was seen on the buccal mucosa, covered by normal-looking mucosa except in one area, which showed a glistening surface representing a probable cystic area (Figure 1A). On palpation, the lesion was ovoid, measuring approximately two cm in diameter, well-demarcated, mobile, and soft to firm in consistency. Regional and cervical lymph nodes were not palpable.

A computed tomography scan showed a well-demarcated tumour nodule in the posterior buccal mucosal area without the involvement of the maxillary bone or sinus region (Figure 1B). Lymph-node involvement was not noted.

A provisional clinical diagnosis of a reactive lesion or salivary gland pathology was given. A detailed clinical evaluation revealed a small cystic area in the mass; hence, a diagnosis of salivary gland tumour was considered and an incisional biopsy was done.

**Figure 2:**
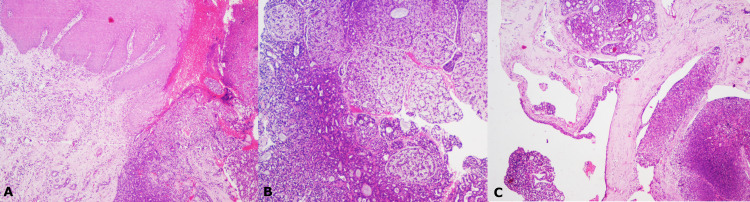
(A) The tumour cells seen close to surface epithelium that exhibits hyperplasia, H&E staining, x40. (B, C) Low power view of the tumour sections showing cells arranged in various patterns, H&E staining, x40.

The microscopic examination of the incisional biopsy specimen showed non-keratinizing stratified squamous epithelium exhibiting hyperplasia and underlying sub-epithelial lesional tissue (Figure 2A). The lesional tissue showed cells arranged in various patterns (Figures 2B, 2C).

**Figure 3:**
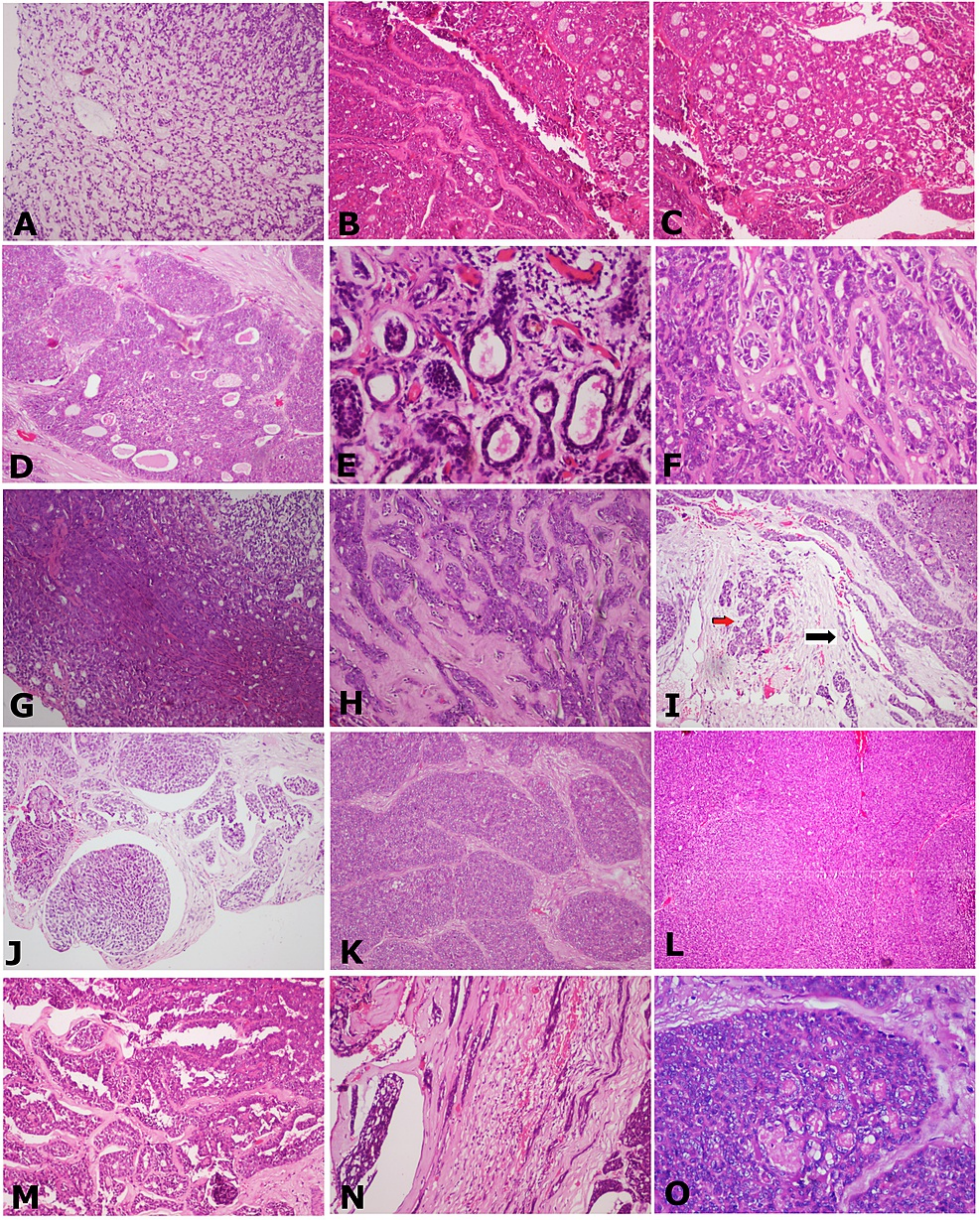
The cells arranged in various patterns such as (A) Filigree, H&E staining, x40. (B) Ribbon and cribriform, H&E staining, x40. (C) Cribriform, H&E staining, x40. (D) Pseudocribriform, H&E staining, x40. (E) Ducts and tubules, H&E staining, x100. (F) Tubular pattern, H&E staining, x40. (G) Layered tubular, H&E staining, x100. (H) Trabecular, H&E staining, x40. (I) Strands (black arrow), nests and islands (red arrow), H&E staining, x40. (J) Glomeruloid, H&E staining x40. (K) Solid organoid, H&E staining, x40. (L) Solid sheet pattern, H&E staining, x40. (M) Papillary cystic, H&E staining, x100. (N) Indian file, H&E staining, x40. (O) Rosette, H&E staining, x100.

The predominant patterns include filigree pattern, ribbon pattern, cribriform, pseudocribriform, ductal, tubular, layered tubular, trabecular, strands, cords, nests, islands, glomeruloid, solid organoid, sheets, papillary-cystic, Indian file pattern, and rosette patterns (Figures 3A-3O).

**Figure 4:**
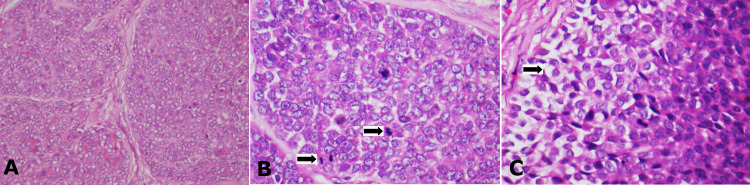
(A) The monotonous tumour cells predominantly showing vesiculated optical clear nuclei, H&E staining, x100. (B) High power view showing cells with vesiculated nuclei and mitotic figures (black arrows), H&E staining, x400. (C) Few of the tumour cells showed clear cell change (black arrow), H&E staining, x400.

The complete tumour mass was made of monotonous cells with poor cytoplasmic borders but with a large clear vesicular nucleus and few nucleoli (Figure 4A). Mitotic figures of around 5-7 per 10 high power fields (HPF) (Figure 4B) and a few clear cells (Figure 4C) were noted in the tumour mass. Tumour islands were seen in the vicinity of blood vessels, but no direct invasion was evident in the vascular or neural elements.

Based on the findings, a diagnosis of PAC, probably high grade, was considered after assessing the presence of numerous mitotic figures.

**Figure 5:**
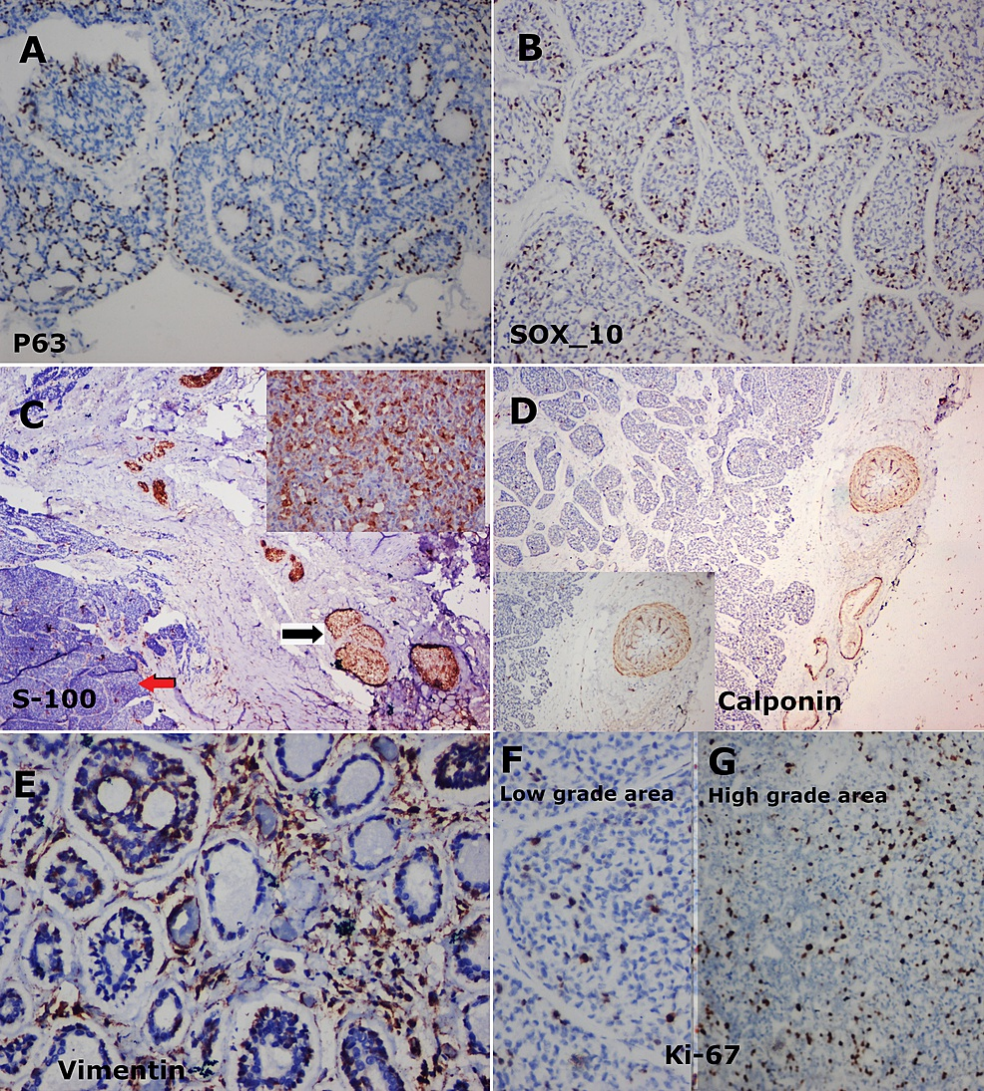
(A) Nuclear expression of p63 protein, patchy in cells of the tumour lobules, IHC staining x200. (B) The tumour cells showed scattered nuclear expression of SOX 10, IHC staining, x40. (C) Tumour cells strongly positive for S-100 (black arrow) and negative in areas of dedifferentiation (red arrow), IHC staining, x40. Inset: High power view shows the tumour cells to be diffusely positive for S-100 in both nucleus and cytoplasm, IHC staining, x400. (D) Near negative expression of calponin in tumour cells, the adjacent smooth muscle wall of the artery shows positivity, IHC staining, x40. Inset: High power view of the same, IHC staining, x200. (E) Few tumour cells and stromal cells show cytoplasmic positivity for vimentin, IHC staining, x200. (F) Low-grade areas of PAC showing 5%-6% Ki-67 positivity. (G) Areas of high-grade transformation showing around 25% to 30% of cells positive for Ki-67, IHC staining, x200.

Hence, IHC was done with a panel of markers such as p63 (Figure 5A), SOX-10 (Figure 5B), S-100 (Figure 5C), calponin (Figure 5D), vimentin (Figure 5E), and Ki-67 (Figures 5F, 5G). The protein p63 showed nuclear positivity and was patchy in distribution among the tumour cells; SOX-10 nuclear positivity was diffusely scattered among tumour cells; S-100 showed nuclear and cytoplasmic positivity in tumour cells in low-grade areas but was completely negative in high-grade areas; calponin was predominantly negative in tumour cells; vimentin was mainly positive in connective tissue cells; and high Ki-67 activity in the range of 25%-30% was seen in high-grade areas (Figure 5G) as compared to low-grade areas (around 5%, Figure 5F).

**Figure 6:**
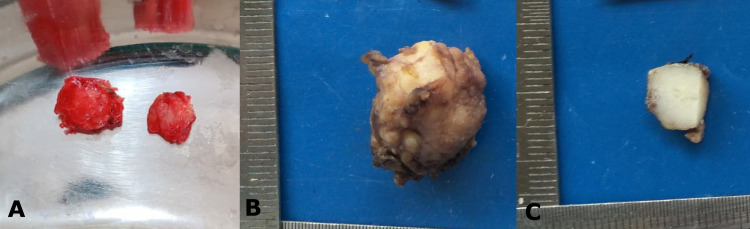
(A) Nodules of tumour mass removed - Surgical grossing - largest measuring 3x2.5 cm. (B) Formalin-fixed specimen of the same. (C) The cut section showed homogenous whitish areas that were soft to firm in consistency.

The above data confirmed the diagnosis of PAC-HGT. The lesion was excised using both an intraoral and extraoral surgical approach and two large lobular tumour masses, approximately 3.0 x 2.5 cm were removed. The cut surface showed a homogenous soft to firm lesional tissue with no evidence of hemorrhage or necrosis (Figures 6A-6C).

**Figure 7:**
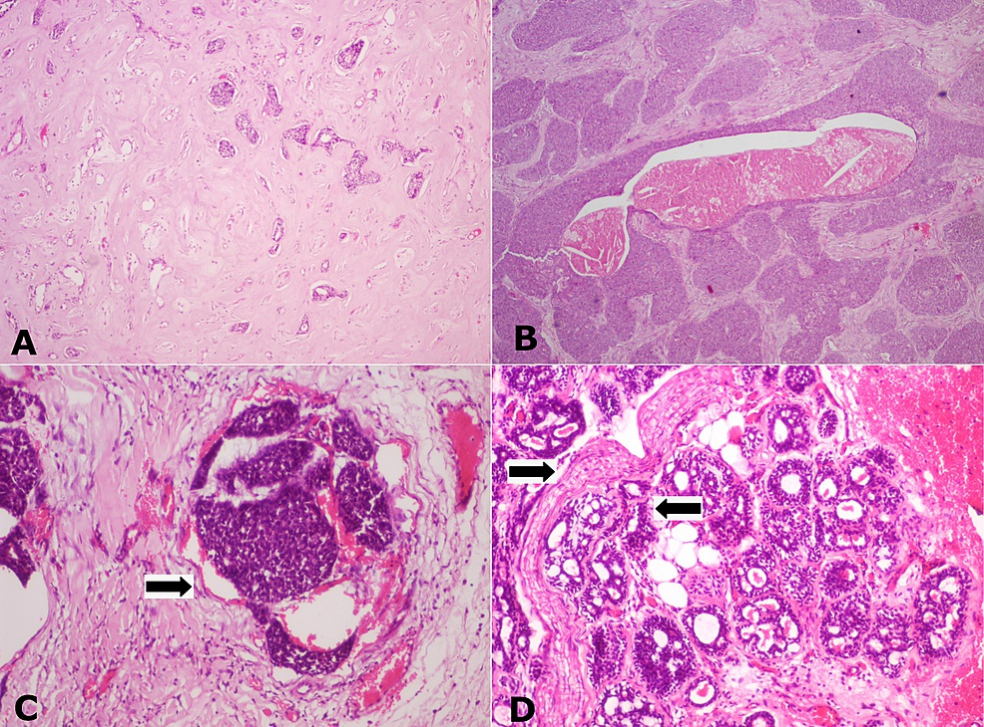
(A) Photomicrograph depicting dense areas of hyalinization entrapping the tumour cells, H&E staining, x40. (B) Solid sheets of tumour cells with areas of central comedo necrosis, H&E staining, x40. (C) Tumour islands invading the blood vessel (black arrow), H&E staining, x40. (D) Tumour islands showing perineural invasion (black arrow), H&E staining, x100.

Histopathological examination of the excised specimen confirmed the presence of various histopathological patterns seen on the incisional biopsy. In addition, the tumour showed a predominance of solid areas in large islands and sheets. Hyalinised nodules and dense areas of hyalinization around tumour islands entrapping the tumour cells were evident (Figure 7A). Many islands showed multiple areas of central comedo necrosis (Figure 7B). Numerous mitotic figures, as high as 5-7 per 10/HPF were noted. Vascular invasion (Figure 7C) and perineural invasion (Figure 7D) was evident. 

An IHC panel of p63, SOX-10, S-100, calponin, vimentin, and Ki-67 was repeated on the excisional mass. The lesional tissue showed similar findings as mentioned before.

The presence of solid areas of tumour cells, comedo necrosis, high mitotic rate, perineural invasion, vascular invasion, and high Ki-67 index prompted us to give a final diagnosis of high-grade PAC.

The surgical wound healing was uneventful on a three-week follow-up. Post-surgical radiation therapy was advised and carried out. On a one-year recall, there is no evidence of recurrence or complications.

AdCC, salivary duct carcinoma, and cribriform adenocarcinoma (CAMSG) were some of the tumours considered for differential diagnosis.

Although a set of common patterns exist and are shared between PAC and AdCC like the tubular, cribriform and solid areas, the presence of various other patterns, like ductal, Indian file, papillary cystic, layered tubular, tubulo-trabecular, and solid sheet patterns, were also noted, giving clues that lead the diagnosis towards PAC. The tumour cells were predominantly monomorphic with a large vesicular nucleus showing nucleoli sticking to the peripheral nuclear wall, unlike the tumour cells of AdCC, which generally show a hyperchromatic, angular nucleus with prominent central nucleoli, hence ruling out the diagnosis of AdCC.

A cystic tumour with no lymph-node involvement at initial presentation and the presence of multiple patterns ruled out the diagnosis of CAMSG.

Multiple areas of comedo necrosis were noted, hence a differential diagnosis of salivary duct carcinoma was considered. The presence of different patterns of cell arrangement, monomorphic cells with a vesicular nucleus, and the absence of a characteristic Roman bridge pattern emphasized a diagnosis of PAC. PAC-HGT is an appropriate diagnosis considering the amount of solid pattern seen with comedo necrosis along with a high mitotic rate in the tissue, reconfirmed by 25%-30% of Ki-67 activity in the tumour cells. [1-7].

## Discussion

Salivary gland tumours of the oral cavity involving the mucous membrane of different areas of the mouth are relatively rare and complex. They form an immensely diverse category of carcinomas. PAC is the second most common intraoral minor salivary gland carcinoma [1,2], which has seen a nosologic expansion related to recent learning of phenotypic, genotypic, behavioural, and morphological variations. According to the WHO 2005 classification, it was called PLGA, a conventional low-grade tumour. The other names given were terminal duct carcinoma and lobar carcinoma [3,4]. The tumour rubs shoulders with variants seeking independent recognition, such as papillary cystadenocarcinoma, low-grade papillary adenocarcinoma (LPASO), and CAMSG, the reprisals of which continues to this day [5,6]. Frequently, it is described as a tumour with variable morphologic appearance, leading to architectural diversity with numerous patterns, but it has a remarkable cytologic uniformity. A characteristic feature of the tumour is nuclear uniformity, with a round to ovoid nuclei and finely dispersed or ground glass type nuclear chromatin classically called the orphan Annie nucleus [7-10].

The tumour is seen in the minor salivary glands of women more frequently (70% of cases), with a male to female ratio of 1:2 and a mean age of 59 years. The common sites of presentation are the palate (32%), soft palate (17%), lip (13%), and buccal mucosa (10%). Around 46% of the malignant minor salivary gland tumours are PAC [9,11,12]. The present case was in a 50-year-old woman on the buccal mucosa close to the maxillary left posterior teeth region, presenting as an asymptomatic mass.

An extravagant spectrum of morphological patterns are seen in PAC, namely; solid, glandular, tubular, trabecular, layered tubular, fascicular stream, cord-like cribriform, filigree, cribriform, ductal, cystic, papillary cystic, targetoid, glomeruloid, and Indian file patterns, but the tumour cell type is monotonous [1,2,4-6]. The presence of a few areas of basaloid cells and clear cells can be encountered [4]. Perineural and perivascular invasion are evident as well. An HGT in the PAC is a rare event, with only a few reported cases till date. Most of the HGT-PACs are related to recurrence and/or radiation. Around seven cases of HGT-PAC have been reported so far in the English literature, arising in the palate (five cases), nasal region (one case), and maxillary alveolus (one case), and only a small percentage are said to be HGT-de novo on first or primary presentation [2,6,11]. HGT and low-grade areas in a PAC can be well-demarcated or partly admixed with each other [2]. PAC with HGT may present with prominent lobular-solid areas; papillary and papillary cystic areas; clear cell differentiation; and comedo necrosis. Cytological features like prominent nucleoli, large nuclei and high mitotic rates are noted [13].

The case we present falls under the rarest category of HGT-PAC. The lesion presented as a cystic tumour on the buccal mucosa with HGT on initial and primary presentation. It is probably the first case of de novo HGT-PAC reported on the buccal mucosa so far, the other de novo HGT-PAC being reported in the palate [2].

The present case showed a well-demarcated tumour mass but did present many small invasive patches. On microscopic examination, the surface epithelium was hyperplastic and the tumour cells were close to the surface epithelium as described in the literature [6]. 

Classic PAC areas with cribriform, pseudocribriform, cords, islands, nests, ribbons, papillary cystic, tubular, layered tubular, trabecular, glomeruloid, single (Indian) file, and sheet patterns were noted. A rare finding of densely hyalinized nodules with numerous small islands of tumour cells undergoing degeneration was seen. Hyalinization can be seen in PAC or AdCC [9]. In the present case, hyalinised areas were mostly interspersed with the HGT areas.

The greater part of the tumour mass consisted of HGT areas (around 50%-60%). The HGT areas presented predominantly as large solid islands with comedo necrosis and cells consisting of large basophilic appearing but vesiculated nuclei in many areas. An increase in normal mitoses of around 5-7 per 10 HPF; perineural and vascular invasion at many sites involving small and large nerve trunks and vessels, respectively was noticed. These features were similar to the cases described by Kikuchi et al. and Simpson et al. [2,11].

Generally, the basaloid salivary tumours include either AdCC, AciCC, basal cell adenoma, or basal cell adenocarcinoma. The present case also appeared as a basophilic tumour as many cells showed an increased nuclear-cytoplasmic ratio with prominent vesicular nuclei. This feature rightly correlates and PACs can be seen as blue or basaloid tumours as described by Sheetala et al. [7,8]. Some reports also document PAC converting to AdCC [14], which invites a judicious use of IHC for confirmation of diagnosis.

The current case did not show any regional lymph node metastasis or distant metastasis on a whole-body scan. Neither was there a history of previous carcinoma or radiation exposure. In cases of PAC, regional and distant metastasis is quite an isolated phenomenon, but the papillary variants of PAC and CAMSGs have a higher preponderance to show metastasis [2,6,11].

An immunohistochemical panel was done to confirm the final diagnosis and HGT. A Ki-67 activity of 25%-30% was seen in the tumour mass spread across every pattern. Calponin was negative in all tumour cells but was taken up by a few connective tissue components. The nominal staining for calponin ruled out myoepithelial cell participation. The p63 was taken up mainly in diffuse patchy areas in the cells of tumour islands. S-100 was strongly and diffusely positive in low-grade areas and almost negative in solid areas showing high-grade differentiation. Vimentin staining was predominantly seen in connective tissue cells. SOX-10 was strong, nuclear positive in large numbers in the tumour cells.

In IHC, the vital marker for a diagnosis of HGT is Ki-67 index. It will be raised on a comparative correlation with conventional PAC areas. Similarly, in our case, the conventional areas showed nearly 5% activity but was markedly raised to 25%-30% in HGT areas. Furthermore, minimal expression of myoepithelial cells was demonstrated with the use of calponin. According to Kikuchi et al. and Simpson et al., numerous other IHC markers can have overlapping expressions; like S-100, CK, vimentin, cyclin D1, p53, p63, oestrogen receptor, androgen receptor, and SOX-10 in cases of PAC, AdCC, and a few other salivary gland tumours [2,11]. Recently use of p40 and CD 117 has been mentioned to get clarity in differentiating between PAC and AdCC [14]. Many instances might call for multiple markers to be used for confirmation in IHC [15].

A classic monomorphic cell type with a vesicular nucleus arranged histologically in numerous patterns along with characteristic IHC expression and valuation using multiple markers helped us to conclude the diagnosis of PAC-HGT as against conventional PAC, AdCC, CAMSG, or other salivary carcinomas.

## Conclusions

Here, we report a rare case of PAC-HGT in the oral cavity, which is probably the first case of PAC-HGT on the buccal mucosa on initial presentation. The tumour presented with a minimum of 15 well-defined histopathological patterns of PAC along with classic HGT areas.

It is very important to have complete clarity on histopathology along with some valuable IHC markers to differentiate between PAC and AdCC, also to differentiate between high-grade PAC and de-differentiated AdCC.

Although extremely rare, the aggressive behaviour of PAC with HGT does exist and needs to be considered as it alters the prognosis and long-term survival of the patient. Such cases add value in understanding, utilizing experience, and IHC for differentiation of PAC in aggressive and high-grade forms. Missing out on this will alter the prognosis and morbidity rates. It is also vital to differentiate between HGT salivary gland tumours as they dictate altered treatment protocols.
